# Impact and effectiveness of Australia's 2025 hybrid RSV immunisation program: results from the PAEDS-FluCAN Network

**DOI:** 10.1016/j.lanwpc.2026.101938

**Published:** 2026-07-28

**Authors:** Ushma Wadia, Peter C. Richmond, Philip N. Britton, Jeremy Carr, Julia E. Clark, Nigel W. Crawford, Te-Yu Hung, Helen S. Marshall, Kristine K. Macartney, Nicholas J. Wood, Brendan J. McMullan, Hannah C. Moore, Paul V. Effler, Allen C. Cheng, Christopher C. Blyth, PAEDS-FluCAN Network, Tom Kotsimbos, Paul M. Kelly, Mark Holmes, Louis Irving, Stephen Vincent, Sanjaya Senenayake, Tony Korman, Caroline Bartolo, Louise Cooley, Peter Wark, Simon Bowler, Jen Kok, Naomi Runnegar, Daniel M. Fatovich, Grant Waterer, Adam W. Bartlett, Anne Kynaston, Joshua R. Francis

**Affiliations:** a The University of Western Australia, Perth, WA, Australia; b The Kids Research Institute Australia, Perth, WA, Australia; c Perth Children's Hospital, Perth, WA, Australia; d The Children's Hospital at Westmead, Sydney, NSW, Australia; e University of Sydney, Sydney, NSW, Australia; f National Centre of Immunisation Research and Surveillance, Sydney, NSW, Australia; g Monash Children's Hospital, Melbourne, Victoria, Australia; h Monash University, Melbourne, Victoria, Australia; i Queensland Children's Hospital, Brisbane, Queensland, Australia; j Royal Children's Hospital, Melbourne, Victoria, Australia; k Murdoch Children's Research Institute, Melbourne, Victoria, Australia; l University of Melbourne, Melbourne, Victoria, Australia; m Royal Darwin Hospital, Darwin, Northern Territory, Australia; n Menzies School of Health Research, Darwin, Northern Territory, Australia; o Women's and Children's Health Network, Adelaide, South Australia, Australia; p Adelaide University, Adelaide, South Australia, Australia; q Sydney Children's Hospital, Sydney, New South Wales, Australia; r The University of New South Wales, Sydney, New South Wales, Australia; s Curtin University, Perth, WA, Australia; t Western Australia Department of Health, Perth, Western Australia, Australia; u Monash Health, Melbourne, Victoria, Australia; v PathWest Laboratory Medicine, Perth, WA, Australia

**Keywords:** RSV, Maternal vaccine, RSV monoclonal antibodies, Effectiveness, Nirsevimab, Children, Hospitalisation, Infant

## Abstract

**Background:**

Australia introduced a hybrid Respiratory Syncytial Virus (RSV) Maternal and Infant Protection Program (RSV-MIPP) in 2025, using National Immunisation Program-funded RSV vaccine for pregnant people from 28 weeks' gestation and jurisdiction-funded nirsevimab. We estimated real-world effectiveness and assessed the impact of the RSV-MIPP program across a participating surveillance network.

**Methods:**

We conducted a national, prospective test-negative case–control study at thirteen PAEDS-FluCAN network hospitals contributing paediatric RSV data. From April 2024 to November 2025, hospitalised children with RSV-associated acute respiratory infection (ARI; cases) and contemporaneous RSV-negative controls were recruited. Immunisation effectiveness was estimated using a test-negative design with multivariable regression, adjusted for prespecified covariates, and calculated as (1−adjusted odds ratio) × 100% with 95% confidence intervals (CIs). Impact was assessed as the 2024–2025 change in the cumulative number of RSV hospitalisations during epidemiological weeks 14–48, excluding hospitals in two states with comprehensive 2024 nirsevimab programs, and stratified by age group.

**Findings:**

Between 1 January and 30 November 2025, 3743 children were recruited (2719 RSV-positive cases; 1024 test-negative controls). Overall effectiveness against RSV-associated ARI hospitalisation in the RSV-MIPP newborn cohort (born from 17 February) was 82·6% (95% CI: 70·6, 89·7). Effectiveness estimates for maternal vaccination and nirsevimab were 81·5% (68·4, 89·2) and 89·6% (73·6, 95·9) respectively. Nirsevimab effectiveness in the catch-up cohort (born 1st October 2024–16th February 2025) was 89·6% (66·4, 96·8). The program's impact, based on a single preprogram comparison year and excluding 2 states, was associated with 43·8%, 20·1% and 8·5% reduction in hospitalisations in infants aged 0–<3 months, 3–<6 months and 6–12 months respectively.

**Interpretation:**

These first southern hemisphere estimates of a hybrid RSV prevention program demonstrated high effectiveness against RSV hospitalisation over a single season. Longitudinal evaluation of the RSV-MIPP is needed to understand sustained effectiveness and year-round population level impacts.

**Funding:**

This work was sponsored by the Australian Government Department of Health, Disability and Ageing and the Australian Centre for Disease Control.

**Research in context**

**Evidence before this study**

Respiratory syncytial virus (RSV) is the leading cause of acute respiratory tract infection among infants and children globally. The morbidity, mortality and economic burden is considerable especially for infants. Maternal RSV vaccination and long-acting monoclonal RSV antibodies (mAb) have demonstrated high efficacy in trials in reducing RSV-associated hospitalisations.

Nirsevimab was shown to be effective in infants in clinical trials across different populations and outcome measures; this has been subsequently confirmed in effectiveness studies in several countries. A recent systematic review and meta-analysis by Sumsuzzman et al. of 32 studies reported overall effectiveness of 83% (95%CI: 77,88) against RSV-related hospitalisations, 81% (71,88) against intensive care unit (ICU) admission and 75% (67,81) against RSV-related lower respiratory tract infection (LRTI) incidence.

Similarly, vaccination in pregnancy (Abrysvo®, Pfizer) has been shown to have high efficacy in preventing infant disease in clinical trials and real world observation data. Data from Argentina and UK suggests that vaccination provides strong protection in early infancy (0–6 months) against RSV-associated LRTI, in particular 0–3 months. Precise point estimates are not available for 3–6 months as yet. Argentinian study by Perez Marc et al. reported sustained protection against RSV-related hospitalisation with vaccine effectiveness (VE) estimates of 71·3% and 68·2% in infants aged 0–3 months and 0–6 months respectively. A recent study by McLachlan et al., using population-level data from Scotland, demonstrated higher VE of 82·9% against RSV-related LRTI hospitalisations in infants aged 0–3 months.

Few countries have taken a hybrid approach, offering both maternal RSV vaccine and nirsevimab in their RSV prevention programs. Recently, population-based observational studies from the USA and France reported the early real-world impact of dual RSV prevention strategies implemented during 2024–2025. The US study demonstrated substantial reductions in RSV-associated hospitalisations among young infants with real-world effectiveness of 70% for maternal vaccine and 81% for nirsevimab. The French study investigated differences between maternal vaccination and nirsevimab within the same population and during the same epidemic period and found nirsevimab was associated with lower risk of RSV-related hospitalisation and severe outcomes including ICU admission, ventilatory support and oxygen therapy compared with maternal vaccination.

**Added value of this study**

Evidence from countries with hybrid RSV prevention strategies remains limited, particularly studies across regions where program design, rollout, and coverage vary. Australia's 2025 RSV Maternal and Infant Protection Program (RSV-MIPP) comprised year-round maternal RSV vaccination delivered through the National Immunisation Program, commencing 3 February 2025, alongside infant nirsevimab made available via state- and territory-based programs. Key programmatic differences included: (i) gestational age for maternal RSV vaccination delivery from 28 weeks' gestation in Australia compared with 32–36 weeks' gestation in the USA, France, and Luxembourg; and (ii) program delivery models, with year-round maternal vaccination implementation in Australia versus seasonal programs in the USA, France, and Luxembourg; (iii) variable immunisation coverage, with higher reliance on nirsevimab reported in the USA and France, and relatively greater use of maternal RSV vaccination in Australia.

Utilising an established national sentinel hospital-based PAEDS-FluCAN surveillance network, we estimated the effectiveness of RSV preventive products and overall impact of the hybrid strategy on targeted paediatric populations following the implementation of the RSV-MIPP. We demonstrate high effectiveness of the RSV-MIPP in infants born after 17 February 2025 at 82% (95% CI: 70·0, 89·2). Individually, immunisation effectiveness of 80·8% (67·8, 88·6) and 89·5% (73·4, 95·8) was estimated for maternal RSV vaccine and nirsevimab respectively. For the RSV-MIPP catchup cohort, nirsevimab effectiveness was 87·3% (63·8, 95·5). For the second RSV season cohort, nirsevimab effectiveness was not demonstrated owing to small numbers in this cohort.

**Implications of all the available evidence**

This is the first published real-world evidence on the 2025 hybrid RSV immunisation program in Australia, with results consistent with findings from the USA despite differences in the setting, gestational age at maternal RSV vaccination and immunisation coverage. Together with assessment of overall impact on RSV associated hospitalisation, this provides important real-world evidence to inform RSV prevention programs in Australia and globally. In the context of growing evidence supporting both infant monoclonal antibody and maternal RSV vaccination, further longitudinal research evaluating the durability, complementarity, and program-level impact of individual and combined prevention strategies across multiple RSV seasons and epidemiological settings is warranted.

## Introduction

Respiratory syncytial virus (RSV) is a leading cause of acute respiratory tract infection, with young children at particular risk of morbidity and mortality.^1^^,^^2^ The incidence of severe RSV disease and hospitalisation is the highest in young infants^3^^,^^4^ and medically at-risk children including those with chronic cardiorespiratory disease, immunosuppression or born prematurely.^5^^,^^6^ In Australia, Aboriginal and/or Torres Strait Islander (hereafter referred as First Nations) children are also at increased risk of severe disease.^7^

Maternal RSV vaccination (Abrysvo®; Pfizer) and long-acting monoclonal antibodies (mAb) (Nirsevimab, Beyfortus®, Sanofi) are registered and available in Australia with additional mAb (Clesrovimab, Enflonsia®, Merck) expected to be licensed in the coming years. Both interventions have demonstrated efficacy in clinical trials in reducing RSV-associated hospitalisation^8^^,^^9^ supported by real-world effectiveness estimates.^10^, ^11^, ^12^, ^13^

Australian states introduced varying nirsevimab programs in 2024 with whole-of-population programs in Western Australia (WA) and Queensland (QLD). Other jurisdictions offered nirsevimab for select high risk populations only. Abrysvo was available on the private market for pregnant people from July 2024 and through a state-funded program in QLD from 1st December 2024 to 2nd February 2025. The national RSV Mother and Infant Protection Program (RSV-MIPP) commenced on 3rd February 2025 with year-round RSV vaccine available for pregnant people from 28 weeks' gestation under the National Immunisation Program (NIP). Nirsevimab was available through state- and territory-funded programs for young infants whose mothers did not receive RSV vaccine or whose mothers were vaccinated <14 days prior to birth, selected infants at higher risk of severe infection (regardless of maternal vaccination status) and children at increased risk of severe infection entering their second RSV season.^14^ In Australia, RSV activity is largely during winter months (April–September) in temperate southern regions but more variable in tropical and northern jurisdictions.^15^ The New South Wales (NSW), Australian Capital Territory (ACT), QLD, Northern Territory (NT), Kimberley and Pilbara regions of WA had year-round nirsevimab programs, whereas other jurisdictions had seasonal programs (commonly April–September). Nirsevimab eligibility largely aligned with the Australian Immunisation Handbook recommendations.^16^ In addition to those with medical risk conditions entering their second RSV season, WA and Victoria recommended nirsevimab for all First Nations children. All but NT had catch-up programs available. For those with catch-up programs, infants <8 months of age were eligible except for NSW and ACT where it was <6 months of age. The programs are summarised in Supplementary Figure S1.

Australia is one of the few countries with a hybrid RSV prevention program, involving maternal vaccination as the primary strategy, with nirsevimab reserved for infants whose mothers were not vaccinated (or vaccinated <14 days before delivery), and select high risk populations. With implementation of the RSV-MIPP, there is a need for a comprehensive strategy assessing coverage, effectiveness and overall impact of these strategies on targeted paediatric populations. Using RSV data from an established national hospital-based surveillance network, we report on the epidemiology of RSV-associated acute respiratory infection (ARI) in children under 2 years of age including risk factors, outcomes, coverage and effectiveness of available RSV prevention program and impact in 2025.

## Methods

Funded by the Australian Government, Paediatric Active Enhanced Disease Surveillance (PAEDS)–Influenza Complications Alert Network (FluCAN) is a national hospital-based network, conducting prospective surveillance for acute respiratory illness in children and adults hospitalised and tested for RSV, influenza and SARS-CoV2.^17^^,^^18^ We conducted a national, test-negative case–control study at PAEDS-FluCAN network hospitals contributing paediatric RSV data (Supplementary Figure S2). Ethics approval was granted by Alfred Health within the National Mutual Acceptance scheme (51,662–154/19; April 2024). A waiver for the need for individual participant consent has been granted as a public health surveillance system.

Analyses are reported according to the Strengthening of Reporting of Observational studies in Epidemiology (STROBE) reporting guideline.^19^

### Study population

Between 1st April 2024 and 30th November 2025, children hospitalised with laboratory-confirmed RSV-associated acute respiratory infection (RSV cases) and contemporaneous test-negative controls were recruited. ARI was defined by acute respiratory symptoms and/or fever. Cases and test-negative controls were identified by screening admission and laboratory records for ARI symptoms and performance of a validated nucleic acid amplification respiratory viral test at the time of admission. To ensure adequate age representation, we established weekly control targets *a priori* at paediatric hospitals: two test-negative controls aged <12 months, two aged 1–<5 years, and one aged 5–<17 years.^18^ No paediatric hospitals are present in two jurisdictions (ACT, TAS).

For the estimation of 2025 RSV immunisation (maternal RSV vaccine or nirsevimab) effectiveness, the population included those enrolled from 1st January to 30th November 2025. To assess impact of the RSV-MIPP on RSV hospitalisations, we compared RSV-associated hospitalisations, stratified by age, occurring during epidemiological weeks 14–48 (1st April–30th November) between 2024 and 2025. The 2024–2025 cases from hospitals in WA and QLD were excluded as both states implemented population-based RSV prevention programmes in 2024.

### Data collection

Demographic and clinical data were systematically collected from the medical records, inclusive of patient case notes, laboratory data and imaging. Variables collected included demographics, medical risk factors, prematurity, symptoms and outcomes for cases and controls. First Nations status was based on self-report from medical records. Medical risk factors assessed included chronic cardiac, respiratory, neurological, renal and hepatic disease, immunosuppression and/or malignancy, diabetes, genetic comorbidities, inborn errors of metabolism and obesity, aligning with existing influenza and COVID-19 surveillance programs.^20^ Nosocomial infection was defined as laboratory testing with a specimen collection date >7 days post admission.

### Immunisation status

Immunisation receipt (including receipt of maternal RSV vaccine for infants <12 months) was determined from the Australian Immunisation Register (AIR) and medical records (including handheld pregnancy records and medication charts).

Eligibility for RSV immunisation (maternal vaccine and/or nirsevimab) in RSV-positive and test-negative children hospitalised with ARI was assessed using date of birth, the presence of medical risk factors and First Nations status (Supplementary Figure S1). Enrolled cases and test-negative controls aged <2 years were divided into four cohorts:

i) RSV-MIPP newborn cohort: born after 17th February 2025 (two weeks after national program commencement, ensuring sufficient time for maternal vaccine protection). Eligible for NIP-funded maternal vaccine or jurisdictional-funded nirsevimab.
ii) RSV-MIPP catch-up cohort: born between 1st October 2024 and 16th February 2025. Eligible for catch-up nirsevimab but also with access to maternal vaccine through private market or Queensland-funded maternal RSV vaccine program (commencing 1st December 2024).
iii) 2nd RSV season cohort: born between 1st October 2023 and 30th September 2024; at least one medical risk factor; or First Nations children in WA and Victoria; and
iv) all other children <2 years of age not eligible for RSV immunisation.

### Statistical analysis

Descriptive analyses of demographics, risk factors, and outcomes were undertaken, first of the overall cohort, then by those eligible for immunisation. Categorical variables were summarised as proportions, while continuous variables reported as medians with interquartile ranges (IQR).

Nosocomial infections and test-negative controls with influenza- or SARS-CoV2-associated ARI hospitalisations were excluded from analyses. RSV cases and test-negative controls with dual immunisation exposure and missing or unknown immunisation status were excluded from effectiveness analysis.

The proportion vaccinated among test-negative controls was used as a proxy for immunisation coverage (nirsevimab or maternal RSV vaccine) and does not represent population-level coverage.

In those with known immunisation status, effectiveness was estimated using a test negative design. Conditional logistic regression was used to estimate odds ratios comparing immunisation status between cases and test-negative controls, conditioning on strata defined by state and epidemiological week. Models were adjusted for age, sex, medical risk factors, prematurity (<37 weeks′gestation), First Nations status, socioeconomic advantage (Socio-Economic Indexes for Area, Australia; SEIFA) and remoteness (Accessibility/Remoteness Index of Australia: ARIA). IE was estimated from the odds ratio of immunisation in RSV cases versus test-negative controls using the formula IE (%) = (1-aOR) × 100%. Model parsimony was assessed using the Akaike Information Criterion (AIC), and models with the lowest AIC prioritised.

To assess relative IE between nirsevimab and maternal RSV vaccine, we utilised the linear combination of estimators function (implemented in Stata as lincom), to estimate the relative odds by comparing the odds of receipt of nirsevimab, compared with odds of receipt of maternal vaccine, adjusted as described above.

To assess the impact of waning immunity, the data were divided by time from immunisation receipt (nirsevimab) to hospitalisation or age at admission (maternal RSV vaccine). Time to event effectiveness was calculated at 0–<3 month, 3–<6 months, ≥6 months.

To explore impact of population differences in those offered maternal vaccination or nirsevimab, effectiveness estimates were repeated restricting analysis among term infants without risk factors. We considered whether misclassification of nirsevimab or maternal vaccination status may have overestimated IE. This was explored by recalculating the odds ratio of exposure assuming imperfect sensitivity of ascertainment, with estimates ranging from 60% to 100%.

To assess the impact of the RSV-MIPP on RSV-associated ARI hospitalisations, the number of cumulative RSV-associated hospitalisations among children under 5 years of age were compared between 2024 and 2025. Age groups most likely to be exposed to RSV-MIPP (0–<3 months; 3–<6 months; 6–<12 months) and age groups less likely to be exposed to RSV-MIPP (1–<2 years and 2–<5 years) were included; WA and QLD were excluded from analyses owing to their infant nirsevimab programs in 2024.

Data analysis was conducted utilising Stata® Software Version 18 (College Station, TX).

### Role of funding source

Study funders did not have any role in the study design, data collection, data analysis, interpretation and writing of the report.

## Results

### Total population

**Fig. 1:**
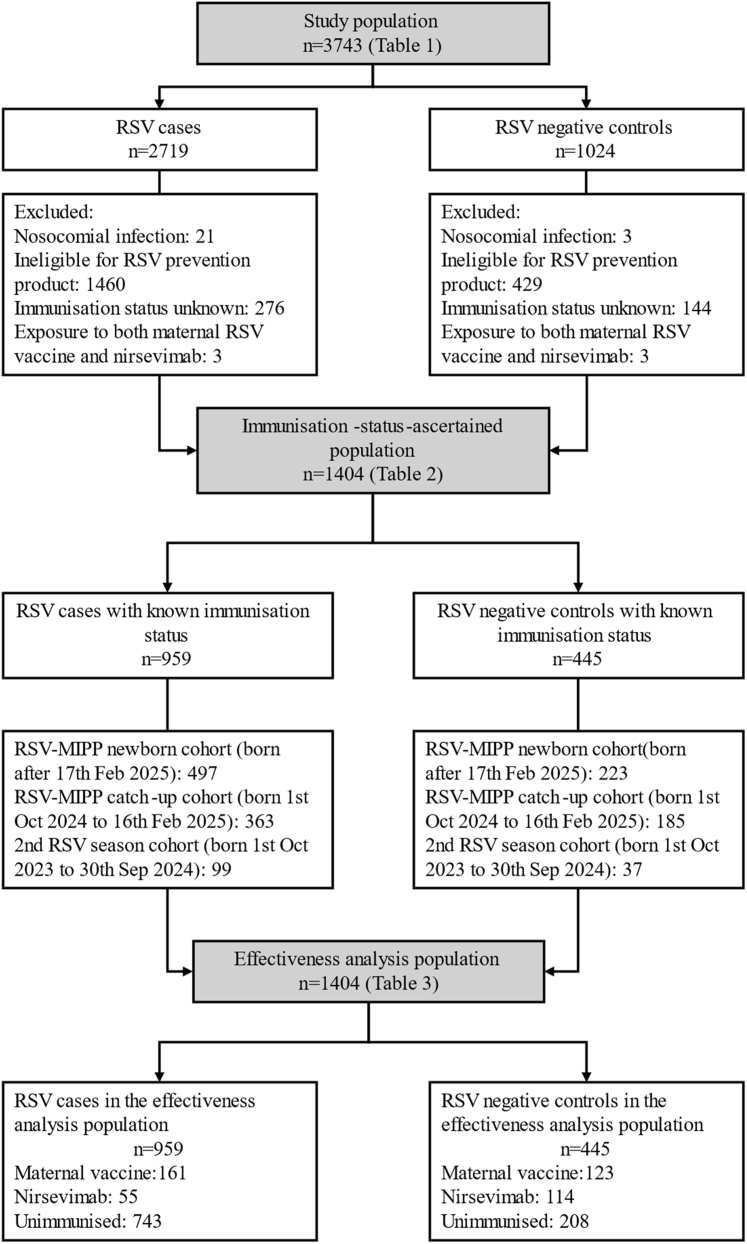
**Study flow diagram**. (RSV-MIPP, RSV Mother Infant Protection Program).

Demographic, risk factors and outcomes for children <2 years of age and hospitalised for an ARI in 2025 were described by RSV cases and test-negative controls status (Table 1). A total of 3743 children <2 years hospitalised in 2025 were identified, including 2719 RSV cases and 1024 test-negative controls (Fig. 1). RSV cases were older (median age 10·92 months; [IQR 4·20, 16·32]) compared to test-negative controls (8·16 months; [IQR 1·92, 14·28]). The peak frequency of admission for RSV-positive cases was epidemiological week 28 (7–13 July 2025; Supplementary Figure S3) with the highest number of RSV cases enrolled in a single month observed in July (epidemiological weeks 27–30).

**Table 1:** Demographic, risk factors and outcomes for hospitalised RSV cases and RSV negative controls aged <2 years of age (total population; n = 3743).

	Case status	
	RSV cases (n = 2719)	RSV negative controls (n = 1024)
**Demographics and risk factors**		
**Age**		
Median age (months; IQR)	10·92 (4·20,16·32)	8·16 (1·92,14·28)
Age group 0–<3 months	518 (19·1%)	311 (30·4%)
Age group 3–<6 months	367 (13·5%)	109 (10·6%)
Age group 6–<12 months	613 (22·6%)	281 (27·4%)
Age group 1–<2 years	1221 (44·9%)	323 (31·5%)
**RSV immunisation cohort**		
RSV-MIPP newborn cohort	644 (23·7%)	309 (30·2%)
RSV-MIPP catch-up cohort	492 (18·1%)	234 (22·9%)
2nd RSV season eligible cohort	115 (4·2%)	52 (5·1%)
All other children <2 years	1468 (54%)	429 (41·9%)
**Male sex**	1544 (56·8%)	605 (59·1%)
**First Nations status**	202 (7·4%)	115 (11·2%)
**Jurisdiction**		
New South Wales	828 (30·4%)	244 (23·8%)
Victoria	761 (28·0%)	272 (26·6%)
Queensland	248 (9·1%)	140 (16·7%)
South Australia	247 (9·1%)	123 (12·0%)
Western Australia	229 (8·4%)	133 (13·0%)
Tasmania^a^	60 (2·2%)	0 (0·0%)
Northern Territory	72 (2·6%)	104 (10·2%)
Australian Capital Territory	274 (10·8%)	8 (0·8%)
**SEIFA** Median (IQR)	4 (3,5)	4 (2,5)
**Prematurity**		
<28 weeks gestation	23 (0·8%)	20 (2·0%)
28–≤32 weeks	47 (1·7%)	25 (2·4%)
32–<37 weeks	192 (7·1%)	99 (9·7%)
Term (>37 weeks)	2457 (90·4%)	880 (86·0%)
**Medical risk factors**	191 (7·0%)	110 (10·7%)
Chronic respiratory disease	60 (2·2%)	51 (5·0%)
Chronic cardiac disease	58 (2·1%)	35 (3·4%)
Chronic neurological disease	51 (1·9%)	18 (1·8%)
Immunosuppression/malignancy	14 (0·5%)	12 (1·2%)
Genetic/metabolic disease	47 (1·7%)	33 (3·2%)
Other^b^	27 (1·0%)	16 (1·6%)
**Nosocomial infection**	21 (0·8%)	3 (0·3%)
**RSV immunisation status**		
Maternal RSV Vaccination only	172 (6·3%)	138 (13·5%)
Nirsevimab only	88 (3·2%)	126 (12·3%)
Maternal RSV vaccination and nirsevimab	3 (0·1%)	3 (0·3%)
**Outcomes**		
Median length of hospital stay (days; IQR)	2 (1, 3)	2 (1, 3)
PICU admission	151 (5·6%)	63 (6·2%)
Non-Invasive (CPAP/BiPAP/High flow>10 L/min)	105 (3·9%)	32 (3·1%)
Intubated	24 (0·9%)	12 (1·2%)
ECMO	0 (0·0%)	3 (0·3%)
Death	0 (0·0%)	2 (0·2%)

IQR, interquartile range; SEIFA, Socio-Economic Indexes for Area, Australia; PICU, Paediatric intensive care unit.

a No RSV negative controls were recruited in Tasmania.

b Includes diabetes, chronic liver disease, chronic renal disease, obesity and other.

Of RSV cases, the majority were born at term with no medical risk factors: 2457 (90·4%) were born ≥37 weeks’ gestation and 191 (7·0%) had medical risk factors (Table 1). Compared to RSV cases, test-negative controls were more likely to be premature (144 [14·1%]) and have underlying medical risk factors (110 [10·7%]). RSV cases had a longer median duration of symptoms prior to admission (3 days [IQR 2, 5] compared to test-negative controls (2 days [IQR 1, 3]).

### Eligible population with known immunisation status

Immunisation eligible infants and children with known immunisation status (n = 1404; Fig. 1) were assessed by overall immunisation status (RSV-MIPP) and both maternal vaccination and nirsevimab status. Of controls, 237/445 (53·3%) were immunised (123/445 [27·6%] maternal RSV vaccine only; 114/445, [25·6%] nirsevimab alone). Use of maternal vaccination was more frequently observed in the RSV-MIPP newborn cohort (of RSV negative controls (Table 2): 161/223 [72·2%] overall; maternal vaccination: 115/223 [51·6%]; nirsevimab: 46/223 [20·6%] compared with the RSV-MIPP catch-up cohort (overall: 70/185 [37·8%]; maternal vaccination: 8/185 [4·3%]; nirsevimab: 62/185 [33·5%]).

**Table 2:** Demographics, risk factors and outcomes by RSV-MIPP, maternal RSV vaccine and nirsevimab status (eligible infants and children with known immunisation status; n = 1404).

	RSV-MIPP status		Maternal RSV vaccine status		Nirsevimab status	
	Immunised (n = 453)	Unimmunised (n = 951)	Immunised (n = 284)	Unimmunised (n = 1120)	Immunised (n = 169)	Unimmunised (n = 1235)
**Case-control status**						
RSV cases	216 (47·7%)	743 (78·1%)	161 (56·7%)	798 (71·3%)	55 (32·5%)	904 (73·2%)
RSV negative controls	237 (52·3%)	208 (21·9%)	123 (43·3%)	322 (28·8%)	114 (67·5%)	331 (32·5%)
**Demographics and Risk factors**						
**Age**						
Median age (months; IQR)	2·53 (1·25, 5·26)	3·97 (1·74, 7·22)	1·87 (1·12, 3·24)	4·22 (1·81, 7·46)	5·88 (2·50, 8·28)	3·19 (1·48, 6·18)
Age group 0–<3 months	256 (56·5%)	394 (41·4%)	205 (72·2%)	445 (39·7%)	51 (30·2%)	599 (48·5%)
Age group 3–<6 months	101 (22·3%)	252 (26·2%)	63 (22·2%)	290 (25·9%)	38 (22·5%)	315 (25·5%)
Age group 6–<12 months	85 (18·8%)	205 (21·6%)	16 (5·6%)	274 (24·5%)	69 (40·8%)	221 (17·9%)
Age group 12–<24 months	11 (2·4%)	100 (10·5%)	0 (0·0%)	111 (9·9%)	11 (6·5%)	100 (8·1%)
**RSVimmunisation cohort**						
RSV-MIPP newborn cohort	335 (74·0%)	385 (40·5%)	265 (93·3%)	455 (40·6%)	70 (41·4%)	650 (52·6%)
RSV-MIPP catch-up cohort	102 (22·5%)	446 (46·9%)	19 (6·7%)	529 (47·2%)	83 (49·1%)	465 (37·7%)
2nd RSV season cohort	16 (3·5%)	120 (12·6%)	0 (0%)	136 (12·1%)	16 (9·5%)	120 (9·7%)
**Malesex**	296 (65·3%)	530 (55·7%)	180 (63·4%)	646 (57·7%)	116 (68·6%)	710 (57·5%)
**FirstNations Status**	70 (15·5%)	81 (8·5%)	22 (7·8%)	129 (11·5%)	48 (28·4%)	103 (8·3%)
**Jurisdiction**						
New South Wales	89 (19·7%)	298 (31·3%)	74 (26·1%)	313 (28·0%)	15 (8·9%)	372 (30·1%)
Victoria	102 (22·5%)	322 (33·9%)	76 (26·8%)	348 (31·1%)	26 (15·4%)	398 (32·2%)
Queensland	84 (18·5%)	62 (6·5%)	35 (12·3%)	111 (9·9%)	49 (29·0%)	97 (7·9%)
South Australia	48 (10·6%)	97 (10·2%)	34 (12·0%)	111 (9·9%)	14 (8·3%)	131 (10·6%)
Western Australia	75 (16·7%)	116 (12·2%)	49 (17·3%)	142 (12·7%)	26 (15·4%)	165 (13·4%)
Tasmania	6 (1·3%)	4 (0·4%)	4 (1·4%)	6 (0·5%)	2 (1·2%)	8 (0·7%)
Northern Territory	46 (10·2%)	38 (4·0%)	11 (3·9%)	73 (6·5%)	35 (20·7%)	49 (4·0%)
Australian Capital Territory	3 (0·7%)	14 (1·5%)	1 (0·4%)	16 (1·4%)	2 (1·2%)	15 (1·2%)
**SEIFA** Median (IQR)	4 (2,5)	3 (2,4)	4 (3,5)	3 (2,4)	3 (1,4)	4 (2,5)
**Prematurity**						
<28 weeks	15 (3·3%)	8 (0·8%)	0 (0%)	23 (2·1%)	15 (8·9%)	8 (0·7%)
28–≤32 weeks	10 (2·2%)	17 (1·8%)	1 (0·4%)	26 (2·3%)	9 (5·3%)	18 (1·5%)
32–<37 weeks	46 (10·2%)	85 (8·9%)	27 (9·5%)	104 (9·3%)	19 (11·2%)	112 (9·1%)
Term	382 (84·3%)	841 (88·4%)	256 (90·1%)	967 (86·3%)	126 (74·6%)	1097 (88·8%)
**Medicalrisk factors**	53 (11·7%)	135 (14·2%)	12 (4·2%)	176 (15·7%)	41 (24·3%)	147 (11·9%)
Chronic respiratory disease	28 (6·2%)	35 (3·7%)	4 (1·4%)	59 (5·3%)	24 (14·2%)	39 (3·2%)
Chronic cardiac disease	23 (5·1%)	45 (4·7%)	7 (2·5%)	61 (5·4%)	16 (9·5%)	52 (4·2%)
Chronic neurological disease	6 (1·3%)	33 (3·5%)	2 (0·7%)	37 (3·3%)	4 (2·4%)	35 (2·8%)
Immunosuppression/malignancy	3 (0·7%)	16 (1·7%)	0 (0%)	19 (1·7%)	3 (1·8%)	16 (1·3%)
Genetic/Metabolic disease	14 (3·1%)	37 (3·9%)	3 (1·1%)	48 (4·3%)	11 (6·5%)	40 (3·2%)
Other^a^	5 (1·1%)	18 (1·9%)	0 (0·0%)	23 (2·1%)	5 (3·0%)	18 (1·5%)
**Outcomes**						
Median length of hospital stay (days; IQR)	2 (1, 3)	2 (1, 3)	2 (1, 3)	2 (1, 3)	2 (1, 4)	2 (1, 3)
PICU admission	44 (9·7%)	72 (7·6%)	21 (7·4%)	95 (8·5%)	23 (13·6%)	93 (7·5%)
Non-Invasive (CPAP/BiPAP/High flow>10 L/min)	28 (6·2%)	54 (5·7%)	10 (3·5%)	72 (6·4%)	18 (10·7%)	64 (5·2%)
Intubated	7 (1·6%)	10 (1·1%)	4 (1·4%)	13 (1·2%)	3 (1·8%)	14 (1·1%)
ECMO	0 (0%)	1 (0·1%)	0 (0%)	1 (<0·1%)	0	1 (<0·1%)
Death	0 (0%)	0 (0%)	0 (0%)	0 (0%)	0 (0%)	0 (0%)

RSV-MIPP, RSV Maternal and Infant Protection Program; IQR, interquartile range; SEIFA, Socio-Economic Indexes for Area, Australia; PICU, Paediatric intensive care unit; CPAP, continuous positive airway pressure; BiPAP, bilevel positive airway pressure.

a Includes diabetes, chronic liver disease, chronic renal disease, obesity and other.

Infants born to RSV immunised mothers were younger (median age 1·87 months; [IQR 1·12, 3·24]) compared to infants born to unimmunised mothers (4·22 [IQR 1·81, 7·46]; Table 2). The median duration from maternal RSV vaccination to birth was 44 days (IQR 29, 62) and to hospital admission was 110 days (IQR 81, 148). Median duration from nirsevimab to hospitalisation was 82 days (IQR 36, 140). Nirsevimab exposed children were more likely to be premature (43 [25·4%]) and have underlying medical risk factors (41 [24·3%]) compared with unimmunised children. Nirsevimab exposed children were more likely to be male (116 [68·6%]), require intensive care unit (ICU) admission (23 [13·6%]) and need respiratory support (CPAP, BiPAP, high flow oxygen or intubation; 21/169 [12·4%]). No other significant differences in demographic, medical risk factors and symptoms were noted by immunisation status.

### Immunisation effectiveness

For infants born after 17th February 2025 (RSV-MIPP newborn cohort), the crude odds of laboratory-confirmed RSV hospitalisation in those born to vaccinated mothers or who received nirsevimab, compared to unimmunised children, was 0·207 (95% CI: 0·147, 0·293). Following adjustment for age, sex, First Nations status, medical risk factors, prematurity, SEIFA and ARIA, grouped by week and state, effectiveness of any RSV immunisation against RSV-associated ARI hospitalisation in the youngest cohort was 82·6% (95% CI: 70·6, 89·7; Table 3). In the same cohort, IE was 81·5% (68·4, 89·2) for maternal RSV vaccine and 89·6% (73·6, 95·9) for nirsevimab. Relative effectiveness was 43·9% (−31·9, 76·0).

**Table 3:** Immunisation effectiveness against RSV hospitalisation (n = 1404; excluding children who received both maternal vaccination and nirsevimab).

Cohort		RSV cases		RSV negative controls		OR (95% CI)	aOR^a^ (95% CI)	Immunisation effectiveness (95% confidence interval)
		Immunised	Unimmunised	Immunised	Unimmunised			
RSV-MIPP newborn cohort (born after 17th Feb 2025; n = 720)	Any immunisation	174	323	161	62	0·207 (0·147, 0·293)	0·174 (0·103, 0·294)	82·6% (70·6, 89·7)
	Maternal vaccination	150	323	115	62	0·250 (0·174, 0·360)	0·185 (0·108, 0·316)	81·5% (68·4, 89·2)
	Nirsevimab	24	323	46	62	0·100 (0·057, 0·176)	0·104 (0·041, 0·264)	89·6% (73·6, 95·9)
	Relative effectiveness					0·400 (0·231, 0·693)	0·561 (0·240, 1·319)	43·9% (−31·9, 76·0)
RSV-MIPP catch-up cohort^b^ (born 1 Oct 2024–16th Feb 2025; n = 548)	Any immunisation	32	331	70	115	0·159 (0·099, 0·254)	0·181 (0·074, 0·442)	81·9% (55·8, 92·6)
	Maternal vaccination	11	331	8	115	0·478 (0·188, 1·217)	0·750 (0·135, 4·170)	25·0% (−>100, 86·5)
	Nirsevimab	21	331	62	115	0·118 (0·068, 0·202)	0·104 (0·032, 0·336)	89·6% (66·4, 96·8)
	Relative effectiveness					0·246 (0·087, 0·694)	0·138 (0·017, 1·115)	85·9% (−11·5, 98·3)
2nd RSV season cohort (born 1st Oct 2023–30th Sep 2024; n = 136)	Nirsevimab	10	89	6	31	0·581 (0·195, 1·729)	1·487^c^ (0·206, 10·734)	NS

RSV-MIPP, RSV Maternal Infant Prevention Program.

a Adjusted by age, sex, medical risk factors, preterm status, First Nations status, SEIFA, ARIA, grouped by week and state of enrolment.

b Maternal vaccine available privately or as part of QLD RSV prevention program from 1st Dec 2024.

c Adjusted by age, sex, SEIFA, ARIA and grouped by month and state of enrolment.

Overall adjusted IE against RSV-associated hospitalisation in the RSV-MIPP catch-up cohort was 81·9% (55·8, 92·6; Table 3). IE was 25·0% (≥100·0, 86·5) for maternal RSV vaccine and 89·6% (66·4, 96·8) for nirsevimab.

No differences in IE estimates were observed when restricting analysis to those without risk factors and those born at term (Supplementary Table S1: Sensitivity analyses restricted to term infants without medical risk factors or born preterm). Insufficient numbers of RSV cases and test-negative controls were recruited to provide reliable estimate in those with risk factors or those born preterm. Achievement of predefined weekly control recruitment targets varied across jurisdictions, ranging from 77·6% in the NT to 97·9% in VIC (Supplementary Table S2A: Control recruitment relative to predefined targets by jurisdiction). Results were similar when analyses were restricted to jurisdictions with consistent recruitment (excluding ACT and Tasmania) and to strata with ≥2 controls per week (Supplementary Table S2B: Sensitivity analysis excluding jurisdictions with small number of controls). IE in the RSV-MIPP catch-up cohort was also similar excluding infants exposed to maternal vaccine as part of Queensland program launched ahead of the national program (Supplementary Table S3: Sensitivity analysis excluding Queensland pre-program maternal RSV vaccination exposures).

Sensitivity analyses correcting for misascertainment found that if the sensitivity of ascertainment was 60%, the true IE would be 72%, and as the sensitivity of ascertainment increased, estimates approached those of the primary analysis (Supplementary Table S4: Sensitivity analysis of immunisation effectiveness assuming imperfect exposure ascertainment).

For the second RSV season cohort, nirsevimab effectiveness was not demonstrated due to small numbers of immunised children. These analyses should be interpreted as exploratory given the limited sample size (Table 3).

In children receiving nirsevimab 0–<3 months prior to hospitalisation, estimated effectiveness was 95·4% (95% CI: 88·4, 98·2) and maintained in those receiving nirsevimab 3–<6 months prior to hospitalisation (66·4% [15·2, 86·7]). Maternal RSV IE was also demonstrated in those aged 0–<3 months and 3–<6 months of age (IE 82·7% [65·9, 91·2] and 91·5% [64·8, 97·9] respectively; Supplementary Table S5: Immunisation Effectiveness against laboratory confirmed RSV hospitalisation by age (maternal RSV vaccination) and by time from immunisation (nirsevimab).

### Impact on RSV-associated ARI hospitalisations of RSV-MIPP

**Fig. 2:**
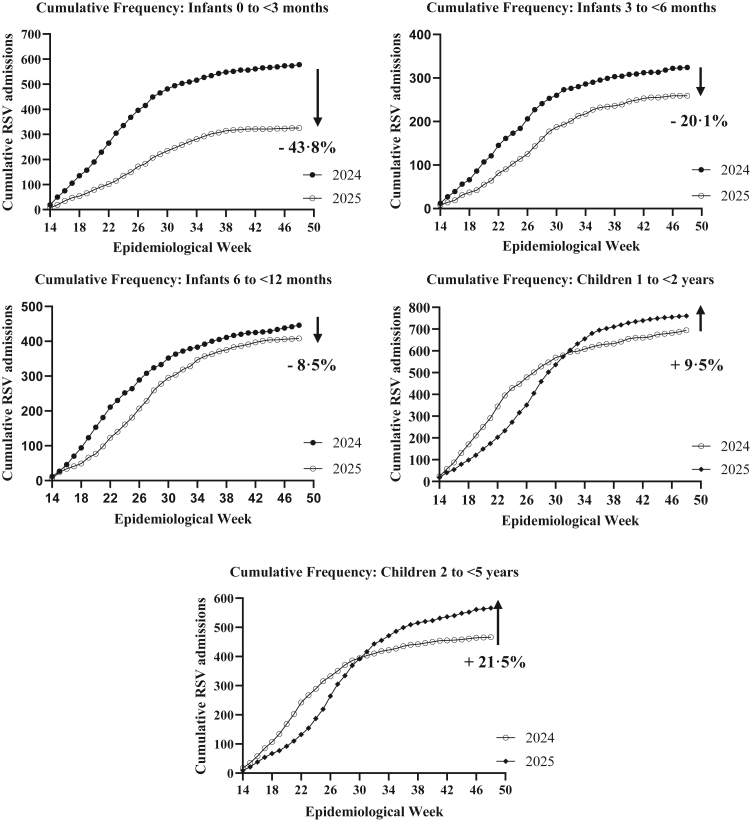
**Cumulative number of respiratory syncytial virus (RSV)-associated hospitalisations of infants and children under 5 years of age within the surveillance network, 2024–2025 (excluding states with population-based programs in 2024)**.

Comparing 2025 to 2024 and excluding hospitals in states with 2024 population-based programs (WA, Queensland), the reduction in RSV cases across the surveillance network was highest at 43·8% in infants aged 0–<3 months (Fig. 2). Reductions were also seen in infants aged 3–<6 months and 6–<12 months of 20·1% and 8·5% respectively. This contrasted with an increase in RSV hospitalisations seen in children aged 1–<2 years (9·5%) and 2–<5 years (21·5%).

## Discussion

This study provides evidence on effectiveness and impact of a hybrid RSV immunisation program utilising maternal RSV vaccine and monoclonal antibody over a single RSV season. Overall, 53·3% of RSV negative controls were immunised, with maternal vaccination favoured in those born after the launch of the national RSV-MIPP. The program reduced RSV-associated ARI hospitalisation in those born from 17th February 2025 with an overall IE of 82·6%. IE was demonstrated for both maternal vaccination and nirsevimab. Nirsevimab effectiveness was also demonstrated in the RSV-MIPP catch-up cohort, but low numbers precluded an assessment in those entering their 2nd RSV season. Effectiveness was observed for up to 6 months of age (for maternal vaccination) or 6 months post-nirsevimab although further conclusions regarding waning effectiveness should be interpreted with caution given low numbers of cases and controls. Compared with the preceding year, RSV-associated hospitalisations were lower among eligible infants, with the highest reduction (43·8%) seen in the youngest infants (0–<3 months). In contrast, children aged 1–<2 and 2–<5 years and unlikely to be eligible for RSV-MIPP, RSV-associated hospitalisations increased by 9·5% and 21·5% respectively reflecting background epidemiological variability.

The estimated effectiveness and impact of the 2025 Australian RSV immunisation program is consistent with 2023–2024 and 2024–2025 northern hemisphere reports (USA, Europe) and 2024 southern hemisphere reports (Chile, Argentina, Australia).^10^, ^11^, ^12^^,^^21^, ^22^, ^23^, ^24^ Most countries offering RSV prevention programs have used either mAb or maternal vaccine with a small number of countries (USA, France, Luxembourg, Australia) using hybrid programs, involving both. USA and France have recently published their 2024–2025 RSV season data. In USA, National Vaccine Surveillance Network (NVSN) data for their 2024–2025 RSV season reported effectiveness of 70% (95% CI: 37, 86) for maternal vaccine (infants younger than 6 months) and 81% (71, 87) for nirsevimab (infants younger than 8 months) against RSV-associated hospitalisations.^22^ In a French study investigating differences between maternal vaccination and nirsevimab within the same population and during the same epidemic period, nirsevimab was associated with lower rates of RSV-related hospitalisation (adjusted HR, 0·74 [95% CI, 0·61, 0·88) and severe outcomes including ICU admission (0·58 [0·42, 0·80]) and need for ventilator support (0·57 [0·40, 0·81]), or requiring oxygen therapy (0·56 [0·38, 0·81]) compared to maternal vaccination.^25^

Key programmatic differences across countries with hybrid programs include: (i) the gestational age for maternal RSV vaccination, commencing from 28 weeks' gestation in Australia compared with 32–36 weeks’ gestation in the USA, France, and Luxembourg (later administration due to potential safety considerations related to earlier gestational vaccination); and (ii) program delivery models, with year-round maternal vaccination implementation in Australia versus seasonal programs in the USA, France, and Luxembourg; (iii) immunisation product coverage with maternal vaccine use higher in Australia compared to France and USA, where nirsevimab coverage was higher.

The broader gestational age window in Australia may influence IE in 2 directions. First, vaccinating earlier from 28 weeks’ gestation increases the number of vaccination encounters, thereby increasing the proportion of women vaccinated well before birth and reducing the number of infants born <14 days after vaccination. Several countries with maternal vaccination programs have reported IE. In infants ≤3 months, the reported effectiveness against RSV-associated LRTI hospitalisation was 78·6% (95% CI: 62·1, 87·9) and 82·2% (75·1, 87·3) in Argentina^12^ and Scotland^26^ respectively. A previous study has suggested that a longer period of up to 5 weeks from maternal RSV vaccine administration to birth is likely to provide higher transplacental transfer of maternal antibodies to the neonate.^27^ This could potentially explain the higher effectiveness estimates reported in Australia and Scotland.

Program delivery models are variable with year-round maternal RSV vaccination implementation in Australia compared to seasonal programs in the USA, France and Luxembourg. We are not yet able to comment on the relative effectiveness of seasonal and year-round maternal vaccination programs due to the lack of complete 12-month data.

France and USA reported higher nirsevimab immunisation coverage compared to maternal vaccination in their studies, in contrast to this study where maternal RSV vaccination coverage was higher than that of nirsevimab. In the French study,^25^ nirsevimab was associated with higher protection against RSV hospitalisation including severe outcomes of ICU admission and respiratory support. We did not observe such findings in our study and our estimated relative effectiveness of nirsevimab compared to maternal vaccination was not significantly different, with wide confidence intervals, and should not be interpreted as evidence of equivalence between interventions. We also observed that nirsevimab exposed children were more likely to require respiratory support or be admitted to ICU. This likely reflects residual confounding, as infants offered nirsevimab are often those with greater underlying medical fragility. Even after adjustment for comorbidities, our analysis may not fully capture the baseline fragility that drives both the likelihood of receiving nirsevimab and the risk of severe illness.

Nirsevimab effectiveness could not be demonstrated in the second RSV season cohort. This analysis is presented for completeness, yet should be interpreted cautiously, as the analysis was based on a small number of vaccinated individuals. Ongoing surveillance and longitudinal follow-up are planned, which will allow accumulation of additional data in this cohort and enable more accurate estimation of effectiveness in future analyses.

Strengths of our study includes systemic collection of clinical, immunisation and laboratory data utilising a national sentinel surveillance network with multiple geographically diverse sites and use of a test-negative design, a robust approach for evaluation of effectiveness that helps minimise bias related to health seeking behaviour.

Our study has several limitations. The observational design may result in a biased IE estimate from unmeasured confounding or incorrect ascertainment of immunisation status or outcome. The potential misclassification and differential missingness of RSV immunisation status arising from differences in how products are delivered and recorded is a limitation. Maternal RSV vaccination is funded through the NIP and is therefore expected to be routinely recorded on immunisation register. In contrast, nirsevimab was delivered through state and territory-based programs with eligibility and delivery pathways varied by jurisdiction. Although nirsevimab doses were required to be recorded in AIR, its jurisdictional recommendation and differing delivery models may lead to incomplete or delayed AIR capture compared with NIP vaccines. To mitigate this, immunisation status was checked again post hospital discharge with AIR and medical records reviewed to validate RSV immunisation exposure for those with missing or unknown status. Despite this, some residual under-ascertainment may persist. This differential ascertainment could bias effectiveness or impact estimates. We have taken this into account by adjustment of potential covariates and conduced sensitivity analyses to account for this. We are not yet able to compare immunisation uptake in our study cohort of controls with population-level vaccine coverage (e.g. from the AIR or national published data), which would have provided additional context on representativeness. The decision to exclude test-negative controls infected with another vaccine preventable respiratory illness (i.e. influenza or SARS-COV2) needed careful consideration as these individuals may be inclined to vaccine acceptance, potentially introducing further biases.

We were unable to collect gestational age at which maternal RSV immunisation was administered. It is hypothesised that the timing of vaccination is likely to influence both the magnitude of maternal antibody response and transplacental antibody transfer potentially impacting on the duration of protection. Inability to collect the gestational age at vaccination limited our assessment of this hypothesis. Few studies that report on maternal effectiveness have taken this into account, providing similar estimates to those we have reported. The median gestational age at vaccination in the Scotland study, where vaccination is also administered from 28 weeks’ gestation, was 31 weeks (IQR: 28, 34) among cases and 30 weeks (28, 33) among matched controls. The maternal RSV immunisation effectiveness was 82·2%.^26^ Unpublished data from the STREETON study looking at maternal immunisation effectiveness in infants ≤6 months across Australian hospitals nationally, reported median gestational age at vaccination was 31 weeks.^28^

The impact evaluation is ecological in nature and therefore, the findings should be interpreted cautiously. However, our results are strengthened by the IE estimates consistent with other studies. The decreased protection and impact with age is consistent, and the absence of effect in children aged 2–<5 years, as negative control, provides complementary context that strengthens the overall interpretation.

Although data were collected from 13 hospitals, the study it is not population-based, and findings may not be generalisable to non-paediatric referral settings. Overrepresentation of more severe cases may limit applicability to milder and community managed cases.

Our study only provides estimates across a single RSV season. Maternal vaccination program commenced in February 2025 (week 5), so likely to have the biggest impact during the peak RSV season (epidemiological weeks 27–30). Longitudinal estimates are needed to fully understand the impact of an all-year round maternal vaccination program and variable nirsevimab program. Lastly, the effectiveness estimates are limited to prevention of RSV-associated hospitalisation and not emergency or primary care presentations. Population-based data are also needed to assess the full impact of the hybrid RSV prevention program.

Despite the limitations, this is the first study estimating IE of a hybrid program in Australia and the southern hemisphere, demonstrating that the hybrid RSV immunisation program is highly effective against RSV-associated hospitalisation in children. Together with assessment of overall impact on RSV-associated hospitalisation, these are important real-world evidence to inform RSV prevention programs in Australia and globally. In the context of growing evidence supporting both infant monoclonal antibody and maternal RSV vaccination, further longitudinal research evaluating the durability, complementarity, and program-level impact of combined prevention strategies across multiple RSV seasons and epidemiological settings is warranted.

## Contributors

All authors attest they meet the ICMJE criteria for authorship.

ACC and CCB designed the study and led recruitment of children with RSV to the network. UW: Data curation and validation, led the analysis and interpretation of data, writing original draft, review and editing for important content. CCB: conceptualisation, methodology, data curation and validation, led the analysis and interpretation of data, writing–review and editing. KKM: funding, governance, data interpretation and presentation, editing manuscript. CCB, UW, PNB, JC, JEC, NWC, TYH, HSM, NJW, and ACC led recruitment at their respective site. CCB, UW, ACC led the analysis and interpretation of data. CCB and UW accessed and verified the data. CCB, UW and ACC were responsible for the decision to submit the manuscript. All authors provided feedback on the draft manuscript and approved publication of the final manuscript.

## Data sharing statement

The datasets generated and/or analysed during the current study are not publicly available but can be made available upon request.

## Editor note

The Lancet Group takes a neutral position with respect to territorial claims in published maps and institutional affiliations.

## Declaration of interests

UW is an investigator on a Sanofi sponsored trial (non-RSV related; no personal renumeration) and Pfizer sponsored trials (non-RSV and maternal RSV vaccine related; no personal renumeration). UW has received travel and accommodation support from Pfizer and Moderna (non-RSV related). PCR has received support to attend international conferences from ReSViNET Foundation and GlaxoSmithKline. PCR has received institutional honoraria for participation in advisory board meetings from Clover Biopharmaceuticals, Pfizer, Astra Zeneca, GlaxoSmithKline, Sanofi and Merck Sharpe & Dohme. PCR is an investigator on Pfizer sponsored trial (maternal RSV vaccine related; no personal renumeration). PNB is an associate investigator on a Sanofi funded pilot project at the Children's Hospital at Westmead; he received a Sanofi speaker's honorarium paid to the Children's hospital at Westmead. JC is an investigator on Pfizer sponsored trial (institutional payments; no personal renumeration). TYH is supported by a NHMRC post graduate scholarship. HSM and ACC are supported by NHMRC investigator Grants. HSM is an investigator on a Sanofi and Pfizer sponsored study with funding provided to her institution (non-RSV related; no personal renumeration). NW is an investigator on a Sanofi, Bionet/Technovalia, Iliad Pharmaceuticals clinical trials (institutional payments; non-RSV related; no personal renumeration) HCM has received institutional honoraria for participation in advisory committees on RSV epidemiology from Merck Sharp and Dohme, Pfizer and Evohealth. HCM is in receipt of research funds from Sanofi-Aventis (Australia) (not related to this study). All other authors declared no conflict of interest.

## Appendix A Supplementary data

Supplemental data clean

PAEDS-FluCAN network authors
