# Supplementary material for: Impact and effectiveness of Australia's 2025 hybrid RSV immunisation program: results from the PAEDS-FluCAN Network
Source: Lancet Reg Health West Pac. 2026 Jul 28;73:101938. doi: 10.1016/j.lanwpc.2026.101938 (PMC13450454; doi:10.1016/j.lanwpc.2026.101938)
Supplement: Supplementary file 1 [file mmc1.docx]

**Supplementary figure 1: Summary of the RSV-Maternal and Infant Protection Program by state and territory 2025**

AIH; Australian Immunisation Handbook

*AIH recommendations: Infants entering their first RSV season who:

- were born to women who did not receive RSV vaccine during pregnancy
- were born within 2 weeks of the mother receiving RSV vaccine during pregnancy
- have risk conditions (see below) for severe RSV disease regardless of maternal vaccination
- were born to mothers with severe immunosuppression, where the immune response to RSV vaccine was impaired
- were born to mothers who have received RSV vaccine in pregnancy but have subsequently undergone a treatment such as cardiopulmonary bypass or extracorporeal membrane oxygenation, that has led to loss of maternal antibodies.

** AIH recommendations: Infants/young children aged 8–24 months entering their second RSV season who have a risk condition (see below) for severe RSV disease, regardless of maternal vaccination or receipt of nirsevimab or palivizumab in their first RSV season.

Risk conditions associated with increased risk of severe RSV disease in infants and young children:

- Preterm birth (<32 weeks gestational age)
- Haemodynamically significant congenital heart disease
- Significant immunosuppression – such as from malignancy, solid organ transplant, haematopoietic stem cell transplant or primary immune deficiencies such as severe combined immunodeficiency
- Chronic lung disease requiring ongoing oxygen or respiratory support
- Neurological conditions that impair respiratory function
- Cystic fibrosis with severe lung disease or weight for length <10th percentile
- Trisomy 21 or another genetic condition that increases the risk of severe RSV disease

**Supplementary figure 2: Sentinel hospitals contributing to paediatric data across the states and territories.**

**Supplementary figure 3: Frequency of eligible RSV-positive hospitalised ARI cases by epidemiological week and age in those <2 years (n=2719; total population).**

**Supplementary table 1: Sensitivity analyses restricted to term infants without medical risk factors or born preterm (n=1205).**

| **Cohort** | | **RSV cases** | | **RSV negative controls** | | **OR**  **(95%CI)** | **aOR***  **(95%CI)** | **Immunisation Effectiveness**  **(95% confidence interval)** |
| --- | --- | --- | --- | --- | --- | --- | --- | --- |
|  |  | **Immunised** | **Unimmunised** | **Immunised** | **Unimmunised** |  |  |  |
| RSV-MIPP newborn cohort  (born after 17^th^ Feb 2025; n=694) | Any immunisation | 164 | 316 | 154 | 60 | 0·202  (0·142,0·288) | 0·177  (0·104,0·302) | 82·3%  (69·8,89·6) |
|  | Maternal vaccination | 142 | 316 | 112 | 60 | 0·241  (0·166,0·349) | 0·187  (0·108,0·323) | 81·3%  (67·7,89·2) |
|  | Nirsevimab | 22 | 316 | 42 | 60 | 0·099  (0·166,0·349) | 0·113  (0·044,0·291) | 88·7%  (70·9,95·6) |
| RSV-MIPP catch-up cohort**  (born 1 Oct 2024 to 16^th^ Feb 2025; n=511) | Any immunisation | 28 | 321 | 54 | 108 | 0·174  (0·105, 0·289) | 0·261  (0·107,0·638) | 73·9%  (36·2, 89·3) |
|  | Maternal vaccination | 11 | 321 | 7 | 108 | 0·529  (0·200, 1·398) | 1·279  (0·178,9·153) | NS |
|  | Nirsevimab | 17 | 321 | 47 | 108 | 0·122  (0·067, 0·221) | 0·166  (0·050, 0·546) | 80·3%  (45·4, 95·0) |

RSV-MIPP RSV Maternal Infant Prevention Program; *adjusted by age, sex, medical risk factors, preterm status, First Nations status, SEIFA, ARIA, grouped by week and state of enrolment; ** maternal vaccine available privately or as part of QLD RSV prevention program from 1^st^ Dec 2024

**Supplementary table 2A: Control recruitment relative to predefined targets by jurisdiction.**

| Jurisdiction† | Controls recruited | Expected Controls^*^ | Target attainment (%) |
| --- | --- | --- | --- |
| NSW | 321 | 384 | 96·4 |
| VIC | 374 | 384 | 97·9 |
| QLD | 191 | 192 | 99·5 |
| WA | 187 | 192 | 97·4 |
| SA | 175 | 192 | 91·2 |
| NT | 149 | 192 | 77·6 |

* Expected controls calculated from predefined recruitment targets of two RSV-negative controls aged <1

and two aged 1–<5 years per week at 8 participating paediatric-admitting hospitals.

† ACT and Tasmania were not paediatric-admitting hospitals and were not subject to predefined recruitment targets.

**Supplementary table 2B: Sensitivity analysis excluding jurisdictions with small number of controls (n=981).**

| **Cohort** | | **RSV cases** | | **RSV negative controls** | | **OR**  **(95%CI)** | **aOR***  **(95%CI)** | **Immunisation Effectiveness**  **(95% confidence interval)** |
| --- | --- | --- | --- | --- | --- | --- | --- | --- |
|  |  | **Immunised** | **Unimmunised** | **Immunised** | **Unimmunised** |  |  |  |
| RSV-MIPP newborn cohort  (born after 17^th^ Feb 2025; n=588) | Any immunisation | 127 | 252 | 149 | 60 | 0·202  (0·140,0·293) | 0·164  (0·094,0·286) | 83·6%  (71·4,90·6) |
|  | Maternal vaccination | 109 | 252 | 115 | 60 | 0·249  (0·169,0·366) | 0·172  (0·096,0·309) | 82·8%  (69·1,90·4) |
|  | Nirsevimab | 13 | 252 | 42 | 60 | 0·077  (0·038,0·154) | 0·078  (0·027,0·220) | 92·2%  (78·0,97·3) |
| RSV-MIPP catch-up cohort**  (born 1 Oct 2024 to 16^th^ Feb 2025; n=393) | Any immunisation | 24 | 203 | 63 | 103 | 0·193  (0·114, 0·327) | 0·287  (0·115,0·718) | 71·3%  (28·2, 88·5) |
|  | Maternal vaccination | 10 | 203 | 7 | 103 | 0·725  (0·268, 1·960) | 1·694  (0·248,11·567) | NS |
|  | Nirsevimab | 14 | 203 | 56 | 103 | 0·127  (0·067, 0·239) | 0·141  (0·041, 0·478) | 85·9%  (52·2, 95·9) |

RSV-MIPP RSV Maternal Infant Prevention Program; *adjusted by age, sex, medical risk factors, preterm status, First Nations status, SEIFA, ARIA, grouped by week and state of enrolment; ** maternal vaccine available privately or as part of QLD RSV prevention program from 1^st^ Dec 2024

**Supplementary table 3: Sensitivity analysis excluding Queensland pre-program maternal RSV vaccination exposures (n=545).**

| **Cohort** | | **RSV cases** | | **RSV negative controls** | | **OR**  **(95%CI)** | **aOR***  **(95%CI)** | **Immunisation Effectiveness**  **(95% confidence interval)** |
| --- | --- | --- | --- | --- | --- | --- | --- | --- |
|  |  | **Immunised** | **Unimmunised** | **Immunised** | **Unimmunised** |  |  |  |
| RSV-MIPP catch-up cohort**  (born 1 Oct 2024 to 16^th^ Feb 2025; n=545) | Any immunisation | 32 | 331 | 67 | 115 | 0·165  (0·104, 0·266) | 0·180  (0·074,0·441) | 82·0%  (55·9, 92·6) |
|  | Maternal vaccination | 11 | 331 | 5 | 115 | 0·764  (0·260, 2·247) | 0·739  (0·133,4·101) | 26·1%  (-310·1, 86·7) |
|  | Nirsevimab | 21 | 331 | 62 | 115 | 0·118  (0·068, 0·202) | 0·104  (0·032, 0·336) | 89·6%  (66·4, 96·8) |

RSV-MIPP RSV Maternal Infant Prevention Program; *adjusted by age, sex, medical risk factors, preterm status, First Nations status, SEIFA, ARIA, grouped by week and state of enrolment; ** maternal vaccine available privately or as part of QLD RSV prevention program from 1^st^ Dec 2024

**Supplementary table 4: Sensitivity analysis of immunisation effectiveness assuming imperfect exposure ascertainment (60–100% estimates).**

| Sensitivity of ascertainment (%) | Any immunisation | | Maternal vaccination | | Nirsevimab | |
| --- | --- | --- | --- | --- | --- | --- |
|  | OR | Immunisation Effectiveness (%) | OR | Immunisation Effectiveness (%) | OR | Immunisation Effectiveness (%) |
| 100 | 0·204 | 79·6 | 0·204 | 79·6 | 0·204 | 79·6 |
| 90 | 0·154 | 84·6 | 0·211 | 78·9 | 0·204 | 79·6 |
| 80 | 0·084 | 91·6 | 0·242 | 75·8 | 0·251 | 74·9 |
| 70 | 0 | 100 | 0·260 | 74·0 | 0·267 | 73·3 |
| 60 | 0 | 100 | 0·279 | 72·1 | 0·280 | 72·0 |

**Supplementary table 5: Immunisation Effectiveness against laboratory confirmed RSV hospitalisation by age (maternal RSV vaccination) and by time from immunisation (nirsevimab).**

| **Cohort** | | **RSV cases** | | | **RSV negative controls** | | | **OR**  **(95%CI)** | **aOR***  **(95%CI)** | **Immunisation Effectiveness**  **(95% CI)** |
| --- | --- | --- | --- | --- | --- | --- | --- | --- | --- | --- |
|  |  | **Immunised** | | **Unimmunised** | **Immunised** | | **Unimmunised** |  |  |  |
| **Effectiveness of maternal RSV vaccination by postnatal age (excluding those receiving nirsevimab)** | | | | | | | | | | |
| All cohorts  (n=1235) | 0 to <3 months  of age | 109 | | 293 | 96 | | 101 | 0·391  (0·274,0·558) | 0·173  (0·088,0·341) | 82·7%  (65·9, 91·2) |
|  | 3 to <6 months  of age | 43 | | 217 | 20 | | 35 | 0·319  (0·176,0·576) | 0·085  (0·021,0·352) | 91·5%  (64·8, 97·9) |
|  | ≥6 months  of age | 9 | | 233 | 7 | | 72 | 0·341  (0·127, 0·915) | 0·681  (0·097; 4·75) | 14·8%  (>-100, 90·3) |
|  | 0 to 6 months  of age | 152 | | 509 | 116 | | 136 | 0·349  (0·257,0·475) | 0·187  (0·111,0·314) | 81·3%  (68·6, 88·9) |
| **Effectiveness of nirsevimab by time from immunisation to RSV hospitalisation (excluding those receiving maternal vaccination)** | | | | | | | | | | |
| All cohorts  (n=1120) | 0 to <3 months  post nirsevimab | 19 | 743 | | 70 | 208 | | 0·076  (0·045, 0·129) | 0·046  (0·018,0·116) | 95·4%  (88·4, 98·2) |
|  | 3 to <6 months  post nirsevimab | 19 |  |  | 28 |  |  | 0·190  (0·104, 0·347) | 0·336  (0·133, 0·848) | 66·4%  (15·2, 86·7) |
|  | ≥6 months  post nirsevimab | 17 |  |  | 16 |  |  | 0·297  (0·148, 0·599) | 0·601  (0·203, 1·780) | 39·9%  (>-100,79·7) |
|  | 0 to 6 months  post nirsevimab | 38 |  |  | 98 |  |  | 0·109  (0·072, 0·163) | 0·105  (0·055, 0·202) | 89·5%  (79·8, 94·5) |

* adjusted by age, sex, medical risk factors, preterm status, First Nations status, SEIFA, ARIA, grouped by week and state of enrolment
