# Supplementary material for: Impact and effectiveness of Australia's 2025 hybrid RSV immunisation program: results from the PAEDS-FluCAN Network
Source: Lancet Reg Health West Pac. 2026 Jul 28;73:101938. doi: 10.1016/j.lanwpc.2026.101938 (PMC13450454; doi:10.1016/j.lanwpc.2026.101938)
Supplement: Supplementary file 2 [file mmc2.docx]

Appendix: PAEDS-FluCAN network authors

| First Name, Middle Name | Surname |
| --- | --- |
| Tom | Kotsimbos |
| Paul M | Kelly |
| Mark | Holmes |
| Louis | Irving |
| Stephen | Vincent |
| Sanjaya | Senenayake |
| Tony | Korman |
| Caroline | Bartolo |
| Louise | Cooley |
| Peter | Wark |
| Simon | Bowler |
| Jen | Kok |
| Naomi | Runnegar |
| Daniel M | Fatovich |
| Grant | Waterer |
| Adam W | Bartlett |
| Anne | Kynaston |
| Joshua R | Francis |
